# Identification of risk factors influencing *Clostridium difficile* prevalence in middle-size dairy farms

**DOI:** 10.1186/s13567-016-0326-0

**Published:** 2016-03-12

**Authors:** Petra Bandelj, Rok Blagus, France Briski, Olga Frlic, Aleksandra Vergles Rataj, Maja Rupnik, Matjaz Ocepek, Modest Vengust

**Affiliations:** Veterinary faculty, University of Ljubljana, cesta v Mestni log 47, 1115 Ljubljana, Slovenia; Institute for biostatistics and Medical informatics, University of Ljubljana, Vrazov trg 2, 1104 Ljubljana, Slovenia; Vodnikova cesta 243, 1000 Ljubljana, Slovenia; Vinharje 6, 4223 Poljane nad Skofjo Loko, Slovenia; National Laboratory for Health, Environment and Food, Prvomajska ulica 1, 2000 Maribor, Slovenia; Faculty of Medicine, University of Maribor, Taborska ulica 8, 2000 Maribor, Slovenia; Centre of Excellence for Integrated Approaches in Chemistry and Biology of Proteins, Jamova cesta 39, 1000 Ljubljana, Slovenia

## Abstract

Farm animals have been suggested to play an important role in the epidemiology of *Clostridium difficile* infection (CDI) in the community. The purpose of this study was to evaluate risk factors associated with *C. difficile* dissemination in family dairy farms, which are the most common farming model in the European Union. Environmental samples and fecal samples from cows and calves were collected repeatedly over a 1 year period on 20 mid-size family dairy farms. *Clostridium difficile* was detected in cattle feces on all farms using qPCR. The average prevalence between farms was 10% (0–44.4%) and 35.7% (3.7–66.7%) in cows and calves, respectively. Bacterial culture yielded 103 *C. difficile* isolates from cattle and 61 from the environment. Most *C. difficile* isolates were PCR-ribotype 033. A univariate mixed effect model analysis of risk factors associated dietary changes with increasing *C. difficile* prevalence in cows (*P* = 0.0004); and dietary changes (*P* = 0.004), breeding Simmental cattle (*P* = 0.001), mastitis (*P* = 0.003) and antibiotic treatment (*P* = 0.003) in calves. Multivariate analysis of risk factors found that dietary changes in cows (*P* = 0.0001) and calves (*P* = 0.002) increase *C. difficile* prevalence; mastitis was identified as a risk factor in calves (*P* = 0.001). This study shows that *C. difficile* is common on dairy farms and that shedding is more influenced by farm management than environmental factors. Based on molecular typing of *C. difficile* isolates, it could also be concluded that family dairy farms are currently not contributing to increased CDI incidence.

## Introduction

*Clostridium difficile* is a spore forming Gram positive anaerobe, which causes hospital and antimicrobial-associated intestinal disease in humans and some animal species. The incidence, severity, and recurrence rates of *C. difficile* infections in humans are increasing [1–6]. Recent prevalence studies suggested that farm animals can be the source for human infection [7–10], which has not been scientifically confirmed [11].

Antibiotic treatment, hospitalization, change of diet and neonatal period were suggested risk factors for *C. difficile* perpetuation in farm and companion animals [12–21]. Most studies investigating the epidemiology of *C. difficile* in bovines were performed on large scale intensive dairy and/or beef operations [8, 11, 22, 23], which is not reflective of the European agriculture. These studies mostly investigated the risk of age [11, 22, 23] or age and antibiotic use [8] for *C. difficile* shedding with feces, many times excluding several possible farm management and environment related risk factors [24].

Family farming is the most common operating farming model in the European Union (EU) [25]. It is strongly supported by the European commission and the majority of member EU states, because of its positive contribution to the socio-economic and environmental sustainability of rural areas [25]. They directly supply the local community and the market in general with products of animal and non-animal origin, within a rich epidemiological environment comprised of people, pet animals, farm animals, wild animals and vermin. To date, there are no studies investigating the epidemiology of *C. difficile* in such an environment. The purpose of this study was, therefore, to determine the prevalence of *C. difficile*, to characterize *C. difficile* isolates and to determine risk factors for *C. difficile* perpetuation within the most common operational farming model in Europe.

## Materials and methods

This study underwent ethical review and was given approval by the National Animal Care Committee at the Ministry of Agriculture, Forestry and Food–Veterinary administration.

### Animal samples

Twenty family dairy farms (Table 1) located in the Slovenian Prealps were included in this study. The average milk yield was 6605.2 L milk/cow/year (3727.32–8876.64 L milk/cow). Diseases recorded and treated on the farms were mostly mastitis, pneumonia, diarrhea, displaced abomasum or other gastrointestinal diseases, ketosis and endometritis/metritis. During the study every herd was checked for infectious diseases, such as paratuberculosis, listeriosis, bovine viral diarrhea and infectious bovine rhinotracheitis (Table 1).

**Table 1:** **Dairy farm characteristics and health status**

Farm characteristics					Health status			
Farm	Housing type	Cattle type	Farm location	No. dairy cows	Paratuberculosis	Listeriosis	BVD	IBR
Farm 1	Free + Grazing	Holstein–Friesian	Rural	17–21	–	–	–	–
Farm 2	Tie	Holstein–Friesian	Rural	11–13	–	–	–	+
Farm 3	Tie	Holstein–Friesian	Village	10–13	–	–	–	+
Farm 4	Tie	Holstein–Friesian	Village	23–27	–	+	–	–
Farm 5	Free	Holstein–Friesian	Village	21–24	–	+	–	–
Farm 6	Tie	Holstein–Friesian	Village	14–18	–	–	–	–
Farm 7	Free	Holstein–Friesian	Village	23–26	–	–	–	–
Farm 8	Tie	Holstein–Friesian	Village	29–33	–	+	–	+
Farm 9	Tie	Holstein–Friesian	Village	16–19	–	–	–	–
Farm 10	Tie	Holstein–Friesian	Village	16–20	–	+	+	+
Farm 11	Tie + Grazing	Simmental	Rural	14–16	–	–	–	–
Farm 12	Tie	Holstein–Friesian	Rural	27–33	–	+	–	–
Farm 13	Tie	Mixed	Rural	16–18	–	–	–	–
Farm 14	Tie	Simmental	Town	11–18	–	+	–	–
Farm 15	Tie	Simmental	Town	13–15	–	–	–	–
Farm 16	Tie	Simmental	Town	13–15	–	–	–	–
Farm 17	Free	Simmental	Village	31–40	–	+	–	–
Farm 18	Tie	Simmental	Rural	11–14	–	+	–	–
Farm 19	Free	Holstein–Friesian	Village	32–37	–	+	–	–
Farm 20	Tie	Simmental	Rural	9–11	–	+	–	–
Total (%)				9–40	0/20 (0%)	10/20 (50%)	1/20 (5%)	4/20 (20%)

BVD: Bovine viral diarrhea, IBR: Infectious bovine rhinotracheitis, Free: Free range.

Products from farms included in this study are mostly sold within the local community, whereas surplus milk and meat are sold to different dairy and meat processing plants in Slovenia, Austria and north-east Italy.

Calves were categorized into three age groups at the time of each sampling (age group one: 0–21 days; age group two: 22–56 days; age group three: 57–180 days), based on their nutritional and digestive physiology [26]. Feces were sampled individually from cows and calves under the age of 6 months in exactly 2 week intervals for a period of 1 year (27 sampling days from November, 2011–November 2012). For all the farms, the same sampling protocol was followed. Samples were taken from the rectum using clean latex gloves (Shield, UK). Cow and calf fecal samples from each farm were pooled separately in the laboratory within 24 h after collection: One gram of fecal sample from each individual was used. Pooled samples were then diluted with sterile saline solution in a 1:3 ratio. The aliquot of 2 mL of every pooled sample, and individual samples from all calves were stored in 2 mL sterile vials (Eppendorf Tubes^®^, Germany) at −70 °C for future analysis.

Heifers and bulls over 6 months of age were excluded from the study because of their limited contact with humans. They are not subjected to significant stress factors of production animals and are usually not used to human handling.

### Environmental samples

Environmental samples were collected from every farm during the meteorological autumn, winter, spring and summer. Manure, silage/hay, water from drinking bowls and soil samples from around the barn were collected in sterile 10–50 mL tubes (Sarstedt, Germany). Samples from other domestic animals present on the farm were collected with sterile swabs (Deltalab, Spain). Barn flies (*Stomoxys calcitrans*) and Barn swallow droppings (*Hirundo rustica*) were sampled only once during the summer. Barn flies were captured alive with hands, using clean latex gloves (Shield, UK) and stored in 10 mL sterile tubes (Deltalab, Spain). Barn swallow droppings were collected from surfaces under the nests within the barn using sterile swabs (Deltalab, Spain).

### Detection of *Clostridium difficile*

All pooled fecal samples were used for molecular detection of *C. difficile* 16S rDNA gene. Samples were processed within 2 days after collection. For total DNA isolation, SmartHelix™ First DNAid kit (IFB, Slovenia) was used as described previously [27]. *Clostridium difficile* 16S rDNA gene was detected using an improved quantitative PCR (qPCR) that has the lowest detection (7.72 CD cells/g feces) and quantification limit (77.2 CD cells/g feces) published to date [27]. Calf fecal samples were analyzed individually when pooled fecal samples tested positive on qPCR.

Pooled fecal samples from cows and individual calf fecal samples, which were positive for *C. difficile* 16S rDNA gene, were then cultured for *C. difficile* [7]. Samples were inoculated into cyloserine-cefoxitin fructose enrichment broth (Oxoid, UK) supplemented with 0.1% sodium taurocholate (Sigma, Aldrich) and cultured for 1 week in anaerobic conditions. Thereafter, 1 mL of inoculated broth from each sample and 1 mL of ethanol were mixed and left for 0.5 h at 20–25 °C. Samples were later inoculated onto standard selective medium enriched with cycloserine and cefoxitin (*C. difficile* agar base and *C. difficile* selective supplement; Oxoid, UK) and left to incubate for 48 h anaerobically at 37 °C. Preliminary identification of isolates was based on typical odor and morphologic criteria. One gram per sample, a swab or one mL of water sediment was used for culture. Environmental samples were cultured as described above.

### Molecular characterization of *Clostridium difficile*

*Clostridium difficile* isolates recovered from fecal and environmental samples were characterized by PCR-ribotyping and toxinotyping. PCR-ribotyping was performed with primers for intergenomic region 16S-23S [28]. Amplification with PCR and electrophoresis of the PCR products on 3% agarose gel were done according to Janezic et al. [29]. PCR ribotypes were named using standard Cardiff/Leeds nomenclature (3-digit code). If reference strains were unavailable, the PCR ribotype was named using keys designated by internal nomenclature. Toxinotyping was performed using subsequent restriction PCR fragments for A3 (part of *C. difficile* toxin gene A, tcdA) and B1 (part of *C. difficile* toxin gene B, tcdB) [30], while the gene for the binary toxin was detected using the protocol described by Stubbs et al. [31].

### Parasite burden on farms

Parasitological evaluations of pooled fecal samples from cows and calves were performed every month during the sampling period using standard flotation and sedimentation techniques [32].

### Data collection and statistical analysis

Information regarding feeding regimens, diseases, and treatments were obtained from farmers, farm veterinary services, and the Central Husbandry Register. Heat index [33] was obtained from the nearest National Meteorological Service weather station (Ljubljana, Slovenia—14°5′E, 46°1′N). A mean value for heat index was calculated over 7 days prior to each sampling day.

The outcome in this study was the presence of *C. difficile* (present, not present) in four subgroups: (1) cows, (2) calves aged 0–21 days (first group), (3) calves aged 22–56 days (second group) and (4) calves aged 57–180 days (third group). The following risk factors were considered for each subgroup: Intestinal parasites, dietary change (a change from conserved to fresh feed), heat index, breed (Holstein–Friesian and Simmental), antibiotic treatment, other treatment (non-antibiotic treatment prescribed by the veterinarian), gastrointestinal disease, mastitis, other diseases, and meteorological season (Tables 4 and 5). The absence of a risk factor was considered as a reference category for odds ratio; a reference category for the outcome “Breed” was Holstein–Friesian.

The analyses were performed at the farm level. First, the univariate assessment of the association between each risk factor and different outcomes was performed by means of logistic regression where farm was included as the random effect. The week of sampling was included in each model as a fixed effect to adjust for the possible confounding effect of time. The variable was treated as continuous and a possible non-linear association was modelled using restricted cubic splines, however none of the models showed a significant effect of the non-linear term as judged by the likelihood ratio test (*p* > 0.05); therefore, only results for the linear association are reported. *P*-values were adjusted with the Benjamini-Hochberg method (P.bh) to control the false discovery rate. Significance level was set to 0.05 for the adjusted *p* values. When estimating the association between heat index and the outcomes a non-linear relation was modeled using restricted cubic splines. None of the models showed a significant effect of the non-linear term as judged by the likelihood ratio test; therefore, only results for the linear association are reported.

Following the univariate assessment, multivariate models were built for each outcome. Backward selection with a Bayes Information Criterion (BIC) cutoff value set to two was used for variable exclusion in random effects logistic regression models. These results were also verified using penalized random effects logistic regression where the penalization coefficient was determined through BIC. The variables selected by the two approaches were very similar; therefore, only results for the backward selection are reported.

Statistical analysis was performed using R language for statistical computing (R version 3.0.1) [34].

## Results

### *Clostridium difficile* prevalence

Between farm prevalence. *Clostridium difficile* was detected in fecal samples from all farms on at least one sampling day (100%). Throughout the year farms were positive for *C. difficile* on an average of 39.8% of sampling dates (whether calves, cows or both). *Clostridium difficile* was identified on each sampling day on at least 3 (15%) and no more than 14 farms (70%).

### Cow prevalence

Fifty-four (54/540; 10%) pooled cow fecal samples were positive for *C. difficile* with qPCR, which ranged from 0–44.4% per farm. Bacterial culture identified *C. difficile* from only one pooled cow fecal sample.

#### Calf prevalence

In calves (*n* = 2442) *C. difficile* 16S gene was detected in 182 pooled samples (182/511, 35.6%), which ranged from 3.7–66.7% per farm. *Clostridium difficile* was identified with qPCR in 243 individual calf samples (243/2442, 10%). Bacterial culture yielded 102 *C. difficile* isolates from 101 calves (Table 2).

**Table 2:** **Results for individual fecal samples from calves and molecular characterization of isolated** ***C. difficile*** **strains**

Samples	Farm	1	2	3	4	5	6	7	8	9	10	11	12	13	14	15	16	17	18	19	20	Overall	%
	No. calves	171	48	97	160	121	69	263	125	48	153	73	98	110	313	83	59	157	70	184	40	2442	100
Analysis	DNA isol.	44	24	6	92	83	26	11	58	2	67	34	6	6	185	62	26	81	41	52	12	918	37.6
	qPCR +	11	8	1	17	32	15	2	15	2	10	9	1	1	30	29	12	24	14	6	4	243	10
	Culture +	2	5	1	8	7	6	2	4	1	5	2	1	0	11	17	7	11	10 (9)	2	0	102 (101)	4.2 (4.1)
Ribotype	Toksinotype																						
001/072	0	1																				1	1
002	0							1	1						1							3	2.9
003	0						1															1	1
005	0			1																		1	1
012	0										3				1							4	3.9
014/020	0							1								1						2	2
018	0									1												1	1
023	IV											1										1	1
033	XIa	1	5		8	7	5				1		1		9	16	7	11	6			77	75.5
071	Tox-																			2		2	2
077	0																		3			3	2.9
SLO 029	0																		1			1	1
SLO 036	0											1										1	1
SLO 084	Tox-								2													2	2
SLO 116	Tox-								1													1	1
SLO 195	0										1											1	1

#### Environmental samples

*Clostridium difficile* was also isolated from 11 winter, 16 spring, 16 summer and 18 autumn environmental samples (Table 3). However, two samples (one from the summer and one from the autumn), which were confirmed positive for *C. difficile*, were later lost during further culture processing. From other domestic animals on sampled farms, only poultry was found positive for *C. difficile*. Stable flies from two farms were found positive for *C. difficile*; only one isolate was then successfully cultured. No *C. difficile* was isolated from fecal droppings from Barn swallows sampled during their peak breeding season.

**Table 3:** ***Clostridium difficile*** **isolates from environmental samples and their molecular characterization**

Environmental samples	*C. difficile* culture results					*C. difficile* strain characterization	
	Winter	Spring	Summer	Autumn	All year	Ribotypes	Toxinotypes
Manure	4/20 (20%)	7/20 (35%)	5/20 (25%)	7/20 (35%)	23/80 (28.7%)	001/072, 002, 014/020, 023, 033, 077, SLO 036, SLO 053, SLO 060	0, IV, XIa, XIc (new)
Soil	5/20 (25%)	8/20 (40%)	7/20 (35%)	8/20 (40%)	28/80 (35%)	001/072, 012, 014/020, 018, 023, 033, 081, SLO 025, SLO 057, SLO 060, SLO 063	0, IV, XIa, XIc (new), tox-
Silage/hay	0/20 (0%)	1/20 (5%)	0/20 (0%)	2/20 (10%)	3/80 (3.75%)	001/072, 003, SLO 116	0, tox-
Water	1/20 (5%)	0/20 (0%)	1/20 (5%)	1/20 (5%)	3/80 (3.75%)	014/020, SLO 036	0, XIa
Other animals on farms	1 (2 strains)/32 (3.1%)–adult rooster	0/33 (0%)	1/24 (4.2%)–rooster 2 weeks	0/26 (0%)	2/115 (1.7%)	045, SLO 060, SLO 196 (new)	V, XIa, tox-
Barn swallows (*Hirundo rustica*)	/	/	0/20 (0%)	/	0/20 (0%)		
Stable flies (*Stomoxys calcitrans*)	/	/	2/20 (10%)	/	2/20 (10%)	033	XIc (new)
Total	11 (12)/112 (9.8%)	16/113 (14.2%)	16/144 (11.1%)	18/106 (17%)	61/475 (12.8%)		

### Molecular characterization

Overall from 103 *C. difficile* strains 16 PCR-ribotypes and 4 toxinotypes were cultured from cows and calves. In cows, only a toxin negative PCR-ribotype 071 was cultured, which was also identified in calves from the same farm. The most predominant *C. difficile* strain in calves was PCR-ribotype 033 (toxinotype XIa; 75.5%). PCR-ribotypes 071, SLO 084 and SLO 116 were toxin negative, whereas ribotype 023 was toxinotype IV. All other ribotypes (001/072, 002, 003, 005, 012, 014/020, 018, 077, SLO 029, SLO 036, SLO 195 new) were toxinotype 0 (Table 2).

Sixty-two *C. difficile* strains grouped into 19 different PCR-ribotypes (one new) and 6 different toxinotypes (toxin negative, 0, IV, V, XIa and XIc-new) were identified in the environment (Table 3). The most predominant *C. difficile* types were SLO 060 and 033 (toxinotype XIa,c). Toxin negative PCR-ribotypes were SLO 057, SLO 116 and SLO 196, whereas PCR-ribotype 023 was toxinotype IV and PCR-ribotype 045 was toxinotype V. All other ribotypes (001/072, 002, 003, 012, 014/020, 018, 077, 081, SLO 025, SLO 036, SLO 053, SLO 063) were toxinotype 0.

PCR-ribotype 001/072 was found in manure, soil and silage, while PCR-ribotype 014/020 was recovered from manure, soil and water samples.

Two strains of *C. difficile* were recovered from an adult rooster (PCR-ribotype/toxinotype; 045/V and SLO 060/XIa) and one new strain was isolated from a two-week-old rooster (SLO 196/toxin negative). Stable flies were infected with *C. difficile* PCR-ribotype/toxinotype 033/XIc (new toxinotype), which was also present in manure and soil samples.

### Parasite burden between farms

Parasites identified in pooled fecal samples were: *Strongylida* (65%), *Paramphistomum cervi* (30%), *Nematodirus* sp. (55%), *Strongyloides* (15%), *Eimeria* sp. (100%), *Monezia* sp. (40%) and *Fasciola hepatica* (5%).

### Univariate analysis of risk factors

In cows (Table 4), the only risk factor associated with *C. difficile* prevalence after adjusting for time of sampling were dietary changes (OR 5.0; 95% CI 2.0–12.1; *P* = 0.0004; P.bh = 0.007).

**Table 4:** **Risk factors associated with the prevalence of** ***C. difficile*** **in cows and third age group of calves (57–180 days)**

Age group	Cows					Calves 57–180 days				
Risk factors	Odds ratio	CI, low	CI, up	*P* value	P.bh	Odds ratio	CI, low	CI, up	*P* value	P.bh
Intestinal parasites cows	0.67	0.27	1.67	0.397	0.685	1.11	0.45	2.73	0.805	0.899
Intestinal parasites calves	0.97	0.47	1.98	0.937	0.976	1.12	0.53	2.37	0.760	0.899
*Dietary change*	*5.00*	*2.06*	*12.11*	0.001	*0.007*	2.84	1.10	7.35	0.030	0.289
Heat index	0.95	0.91	0.99	0.046	0.323	0.98	0.93	1.02	0.423	0.899
Breed	1.80	0.58	5.57	0.304	0.685	2.82	1.23	6.47	0.014	0.268
Antibiotic treatment: cows	1.72	0.86	3.43	0.118	0.323	1.26	0.59	2.69	0.545	0.899
Other treatment: cows	1.85	0.91	3.74	0.087	0.323	1.16	0.51	2.60	0.715	0.899
GI diseases: cows	1.60	0.54	4.68	0.390	0.685	0.86	0.18	4.18	0.862	0.909
Mastitis	1.30	0.58	2.88	0.513	0.750	1.16	0.48	2.78	0.736	0.899
Other diseases: cow	3.38	0.72	15.73	0.119	0.323	0.00	0.00	∞	1.000	1.000
Antibiotic treatment: calves	0.64	0.19	2.11	0.468	0.741	0.56	0.12	2.64	0.472	0.899
Other treatment: calves	1.18	0.46	3.02	0.723	0.858	1.26	0.44	3.61	0.664	0.899
GI diseases: calves	0.00	0.00	∞	0.976	0.976	1.55	0.16	14.32	0.696	0.899
Other diseases: calves	1.40	0.42	4.66	0.5764	0.782	1.33	0.35	5.06	0.669	0.899
Meteorological season-winter vs				0.060	0.323				0.230	0.899
Spring	1.20	0.48	3.00	0.693	0.858	1.52	0.57	4.01	0.395	0.899
Autumn	0.55	0.14	2.10	0.391	0.685	0.58	0.14	2.36	0.451	0.899
Summer	0.32	0.09	1.14	0.079	0.323	0.64	0.17	2.34	0.503	0.899

CI: 95% confidential intervals.

P.bh: *P* values adjusted with Benjamimi and Hochberg method.

In the first age group of calves (Table 5) risk factors increasing *C. difficile* prevalence were dietary changes (OR 5.08; 95% CI 2.3–77.9; *P* = 0.004; P.bh = 0.04) and breeding Simmental cattle (OR 5.3; 95% CI 1.9–14.7; *P* = 0.001; P.bh = 0.03).

**Table 5:** **Risk factors associated with the prevalence of C. difficile in calves—First (0–21** **days) and second (22–56** **days) age group**

Age group	Calves 0–21 days					Calves 2–56 days				
Risk factors	Odds ratio	CI, low	CI, up	*P* value	P.bh	Odds ratio	CI, low	CI, up	*P* value	P.bh
Intestinal parasites cows	0.61	0.27	1.37	0.237	0.612	1.18	0.50	2.82	0.694	0.879
Intestinal parasites calves	0.71	0.36	1.38	0.322	0.612	1.71	0.83	3.52	0.143	0.543
*Dietary change*	*13.27*	*2.26*	*77.93*	0.004	*0.039*	1.19	0.31	4.52	0.793	0.941
Heat index	1.00	0.96	1.04	0.866	0.951	1.00	0.96	1.05	0.668	0.879
*Breed*	*5.27*	*1.88*	*14.76*	0.002	*0.029*	2.77	0.87	8.83	0.083	0.395
*Antibiotic treatment: cows*	1.94	0.54	1.02	0.929	0.951	*6.62*	*1.45*	*3.10*	0.003	*0.032*
Other treatment: cows	1.56	0.79	3.06	0.197	0.612	2.60	1.21	5.59	0.014	0.088
GI diseases: cows	2.69	0.34	0.96	0.951	0.951	1.38	0.39	4.82	0.608	0.879
*Mastitis*	1.09	0.51	2.29	0.819	0.951	*3.48*	*1.52*	*7.94*	0.003	*0.032*
Other diseases: cow	7.57	0.38	1.70	0.486	0.768	1.16	0.20	6.69	0.865	0.966
Antibiotic treatment: calves	1.03	0.41	2.54	0.943	0.951	1.94	0.72	5.20	0.188	0.556
Other treatment: calves	1.14	0.50	2.57	0.745	0.951	3.98	0.59	1.54	0.371	0.767
GI diseases: calves	1.76	0.38	8.08	0.463	0.768	0.00	0.00	∞	0.988	0.994
Other diseases: calves	0.54	0.16	1.76	0.311	0.612	1.00	0.31	3.22	0.994	0.994
Meteorological season—winter vs				0.089	0.423				0.383	0.767
Spring	2.02	0.84	4.84	0.114	0.431	0.71	0.27	1.87	0.498	0.859
Autumn	1.66	0.63	4.36	0.300	0.612	0.61	0.20	1.90	0.404	0.767
Summer	3.35	1.22	9.18	0.018	0.116	1.27	0.40	4.02	0.682	0.879

CI: 95% confidential intervals, P.bh: *P* values adjusted with Benjamimi and Hochberg method.

Antibiotic treatment (OR 3.1; 95% CI 1.4–6.6; *P* = 0.003; P.bh = 0.03) and mastitis (OR 3.4; 95% CI 1.5–7.9; *P* = 0.003; P.bh = 0.03) increased *C. difficile* prevalence in the second age group of calves (Table 5).

No risk factors associated with the *C. difficile* prevalence in the third age group of calves (Table 4) were identified after adjusting for time of sampling.

### Multivariate analysis of risk factors

In cows, dietary changes were associated with the prevalence of *C. difficile* (OR 5.8; 95% CI 2.4–14.4; *P* = 0.0001). Similarly, in the first age group of calves, dietary changes were associated with the prevalence of *C. difficile* (OR 17.2; 95% CI 2.8–106.0; *P* = 0.002). Mastitis was identified as a risk factor in the second group of calves (OR 1.6; 95% CI 0.7–3.4; *P* = 0.001). Dietary changes also increased the prevalence of *C. difficile* in the third group of calves (OR 2.8; 95% CI 1.0–7.4; *P* = 0.03) (Table 6).

**Table 6:** **Multivariate analysis**

Outcome		Regression coefficient	Odds ratio	CI, low	CI, up	*P* value
Cows	Intercept	−2.490				<0.0001
	*Dietary change*	*1.772*	*5.881*	*2.401*	*14.405*	*0.0001*
	Intestinal parasites cows	−0.448	0.639	0.248	1.644	0.3531
Calves	Intercept	−0.437				0.2357
First age group	*Dietary change*	*2.849*	*17.263*	*2.810*	*106.027*	*0.0021*
	Intestinal parasites calves	−0.430	0.650	0.330	1.282	0.2145
Calves	Intercept	−1.909				<0.0001
Second age group	*Mastitis*	*1.407*	*4.083*	*1.729*	*9.639*	*0.0013*
	Intestinal parasites calves	0.488	1.629	0.773	3.432	0.1991
Calves	Intercept	−2.576				<0.0001
Third age group	*Dietary change*	*1.037*	*2.819*	*1.073*	*7.407*	*0.0354*
	Intestinal parasites calves	0.061	1.062	0.502	2.246	0.8739

Calves first age group: 0–21 days; Calves second age group: 22–56 days; Calves third age group: 57–180 days.

Parasites were not shown to be a risk factor, which would directly influence the prevalence of *C. difficile*. However, they were identified to influence risk factors, which increased the prevalence of *C. difficile* in the multivariate analysis (Table 6).

## Discussion

This study investigated the role of family farming on the ecology and epidemiology of *C. difficile*, which could be associated with the community-acquired CDI [7–10]. Dietary changes were the most prominent risk factor associated with the prevalence of *C. difficile*. The *Clostridium difficile* ribotypes identified in this study suggest that family dairy farming in Europe is an unlikely source for CDI.

Community-acquired CDI is a significant medical problem in human medicine. Animal contact is suggested as a potential risk factor for the development of community-acquired CDI [35, 36], because of the high prevalence of *C. difficile* in pigs, cattle and poultry on large scale intensive farms [8, 11, 20, 21, 35–38]. Intensive farming management subjects animals to a substantial stress, which increases the likelihood for pathogen transmission [39]. Human animal interaction in large intensive farms is reduced to a minimum and animals in intensive production have limited contact with other animal species that could harbor or transmit pathogenic organisms. Most likely transmission of a pathogen from large intensive farms, therefore, is through a food chain. Farming management on smaller family farms is less stressful for animals and has smaller negative impact on the environment [40]. Such farms are also more interlinked within the community, and a direct or indirect transmission of pathogens between animals, and animals and humans, is possible, including food chain transmission [41].

Several longitudinal studies investigated the prevalence of *C. difficile* in different domestic animal species during different ages or production stages, spanning over a period of 1 month to a year [11, 21–23, 38, 42]. The farm prevalence in this study varied from 3.7 to 74.1%; all farms were positive on at least one sampling day. Other studies also suggest transient shedding patters of *C. difficile* [11, 42]. A prevalence of 10% in cows was found in this study. This is more than in large intensive dairy farms where prevalence of 0.95 [10], 1.5 [43], 2.4 [44] and 4.5% [45] were reported based on a single sampling interval. As expected, calves (35.7%) had much higher *C. difficile* prevalence than cows in this study. Studies reporting prevalence of *C. difficile* in calves reported prevalence from 6 to 22% [10, 22–24, 43, 46, 47], and even 56% in calves less than 7 days old [36]. The use of qPCR as a screening method made *C. difficile* detection more sensitive [27], which most likely accounted for the higher *C. difficile* prevalence in this study compared to other studies. Several sampling stages over a prolonged period are also more likely to identify bacteria in the investigated population.

*Clostridium difficile* bacterial culture results in this study, however, are more in line with previously published data. Considering the results of a bacterial culture, the prevalence in cows and calves would be 0.2% (1/540 pooled samples) and 4.1% (101/2442 individual samples), respectively. Results based on the bacterial culture indicated lower *C. difficile* prevalence than that reported in studies investigating animals on large intensive farms [8, 10, 22, 23, 37, 48, 49].

Sixteen PCR-ribotypes were identified from cattle samples and 19 from environmental samples. Eleven PCR-ribotypes were found in both, cattle and the environment. Two new PCR-ribotype strains (SLO 195, SLO 196) and a new toxinotype (XIc) were identified. PCR-ribotype 033 was the most frequently determined PCR-ribotype in this study, which has often been reported in calves and humans, but has not been linked to the community-acquired CDI [7, 10, 23, 43, 46, 50, 51]. Other PCR-ribotypes found were associated with CDI including ribotypes 001/072, 002, 012 and 014/020 [10, 29, 52, 53]. PCR ribotype 014/020 was previously isolated from meat products in Canada [54, 55]. PCR ribotype 078, which is closely associated with the rising incidence of community-acquired CDI [5, 10, 52] and has been identified on large cattle farms [8, 23, 48–50, 56], has not been detected in this study. It has been suggested that the exposure to less toxigenic strains of *C. difficile* such as PCR-ribotype 033 may help protect people or animals against more toxigenic strains and decrease the incidence of community-acquired CDI [57, 58].

The most important risk factor influencing the prevalence of *C. difficile* in this study were dietary changes. A similar result was found in horses [5, 13, 18] but not in other farm animals. Breeding/rearing Simmental cattle increased the risk for *C. difficile* shedding in the first age group of calves. They were at least five times more likely to shed *C. difficile* than Holstein–Friesian calves in the same age group. We were not able to identify the reason for this prevalence difference. Most *C. difficile* ribotypes identified in Simmental cattle were not regarded as dangerous for CDI, which could competitively reduce the presence of more dangerous strains of *C. difficile* in the environment and potentially make them a safer breed of cattle [57, 58].

The prevalence of *C. difficile* in cows was not associated with the presence of diseases, nor with antibiotic and non-antibiotic treatment, which is in agreement with previous studies [24, 48, 49]. *Clostridium difficile* prevalence in the second age group of calves, however, was sensitive to the antibiotic treatment in cows, as well as to the presence of mastitis on the farm. Possible reason for this finding is the shift in rumen microbiota in calves after the age of 3 weeks [26]. Another reason is a greater likelihood of the second age group of calves to be fed waste milk [59].

Gastrointestinal diseases were not linked to increased prevalence of *C. difficile* in cows or calves. Most animals included in the gastrointestinal disease group in this study had diarrhea. Diarrhea was [19, 37] or was not [24, 60–62] identified as a risk factor in other studies. Intestinal parasites have contributed to the potency of risk factors identified in the multivariate analysis. Interaction between intestinal pathogens is often the culprit for the development of gastrointestinal diseases in individuals and the population [63, 64] and warrants detailed investigation in the population.

Environmental temperatures and humidity are considered significant stress factors for production animals, which can influence the presence of *C. difficile* in feces [65–68]. Rodriguez-Palacios et al. [55] reported a positive association between *C. difficile* isolation in meat products in Canada with the months of January and February. In calves aged less than 1 month it was more likely to isolate *C. difficile* from their feces during the months of May, June and July when compared to August [24]. In the present study meteorological season and heat index did not influence the prevalence of *C. difficile*.

It is always important to be familiar with factors, which may influence the epidemiology of the disease and the biology of the etiological factor. This study provides significant information with regards to the epidemiology of *C. difficile* on the most prominent farming model in Europe. The results of this study indicate that it is unlikely that mid-size family dairy farms in Europe harbor highly pathogenic *C. difficile* strains, which are found to cause disease in animals and humans. The predominant presence of the benign *C. difficile* PCR-ribotype 033 may even have a protective rather than pathologic role in the pathogenesis of the disease.

## Abbreviations

BVD: bovine viral diarrhea
CDI: *Clostridium difficile* infection
IBR: infectious bovine rhinotracheitis
MAP: *Mycobacterium avium* subsp. *paratuberculosis*
